# Primary Cardiac Angiosarcoma: A Review

**DOI:** 10.7759/cureus.41947

**Published:** 2023-07-16

**Authors:** Naina Kumari, Sagar Bhandari, Anzal Ishfaq, Samia Rauf R Butt, Chukwuyem Ekhator, Amanda Karski, Bijan Kadel, Mohamedalamin Alnoor Altayb Ismail, Tenzin N Sherpa, Ahmed Al Khalifa, Bashar Khalifah, Nhan Nguyen, Slobodan Lazarevic, Mohammad Uzair Zaman, Ashraf Ullah, Vikas Yadav

**Affiliations:** 1 Internal Medicine, Dow Medical College, Dow University of Health Sciences, Karachi, PAK; 2 Cardiology, Tucson Medical Center, Tucson, USA; 3 Internal Medicine, Mayo Hospital, Lahore, PAK; 4 General Practice, California Institute of Behavioral Neurosciences and Psychology, Fairfield, USA; 5 Neuro-Oncology, New York Institute of Technology College of Osteopathic Medicine, Old Westbury, USA; 6 Emergency Medicine, American University of Antigua, Miami, USA; 7 Internal Medicine, Nepal Medical College and Teaching Hospital, Kathmandu, NPL; 8 Internal Medicine, Ibrahim Malik Teaching Hospital, Khartoum, SDN; 9 Internal Medicine, Kathmandu University, Nepal Medical College, Kathmandu, NPL; 10 Medicine, College of Medicine, Sulaiman Alrajhi University, Al Bukayriyah, SAU; 11 Pharmacy, Al Qassim National Hospital, Buraydah, SAU; 12 Internal Medicine, University of Debrecen, Debrecen, HUN; 13 Internal Medicine, Faculty of Medicine, University of Nis, Nis, SRB; 14 Medicine, Bacha Khan Medical College, Mardan, PAK; 15 Internal Medicine, Pandit Bhagwat Dayal Sharma Post Graduate Institute of Medical Sciences, Rohtak, IND

**Keywords:** tumor, adult cardiac surgery, primary cardiac angiosarcoma, angiosarcoma, cardiology

## Abstract

Primary cardiac angiosarcoma is a rare and aggressive malignancy originating from the endothelial lining of cardiac blood vessels. This review covers various aspects of the disease, including its pathogenesis, clinical presentation, diagnosis, treatment, and prognosis. The primary characteristic of cardiac angiosarcoma is the rapid growth of abnormal blood vessels that invade the heart muscle, leading to the destruction of healthy tissue. Due to its infiltrative nature and early spread, diagnosing and treating cardiac angiosarcoma present significant challenges. Transesophageal echocardiography (TEE) plays a crucial role in diagnosing cardiac tumors such as angiosarcoma due to its high sensitivity. Additional imaging techniques such as computed tomography (CT) and cardiac magnetic resonance imaging (MRI) help assess tumor anatomy and identify metastases. Histopathological examination and immunohistochemistry are essential for confirming the diagnosis, as they reveal distinct histological features and specific endothelial markers associated with primary cardiac angiosarcoma. Targeted therapies directed at the angiogenic mechanisms and molecular abnormalities hold promise for improving treatment outcomes. Early detection of primary cardiac angiosarcoma remains challenging due to its rarity, and the prognosis is generally poor due to advanced disease at the time of diagnosis. The review emphasizes the importance of a multidisciplinary approach and collaboration among different specialties to optimize the diagnosis, treatment, and follow-up care of patients with primary cardiac angiosarcoma. The ultimate goal is to enhance diagnostic methods and therapeutic approaches by advancing knowledge and promoting further research into this aggressive malignancy.

## Introduction and background

Cardiac angiosarcoma is an uncommon malignant tumor arising from the endothelial cells (ECs) lining the blood vessels of the heart and accounts for approximately 25%-30% of all primary cardiac malignancies. It is considered to be the most fatal and aggressive primary cardiac malignancy. Primary cardiac angiosarcoma arises directly within the heart, unlike secondary cardiac tumors that originate from elsewhere in the body [1]. While it predominantly affects the right side of the heart, particularly the right atrium, it can also impact other cardiac chambers and structures. The characteristic feature of cardiac angiosarcoma is the rapid formation of abnormal blood vessels that invade the myocardium, leading to the destruction of healthy heart tissue. Due to its infiltrative nature and early metastasis, cardiac angiosarcoma poses significant challenges in both diagnosis and treatment.

Realdo Columbus made the first mention of this aggressive malignancy in 1559. The first clinical diagnosis of a primary cardiac sarcoma, however, was not reported until 1934 [2]. With an autopsy incidence of 0.0001%-0.030%, or around one in every 500 cardiovascular surgery patients, primary cardiac neoplasms are extremely uncommon [3,4]. Only 25% of these tumors are malignant, with the majority being benign. Cardiovascular sarcomas make up 95% of cases in the malignant category, and primary cardiac angiosarcoma, which accounts for 30% of these instances, is the most frequent histological subtype [5]. Male predominance (2-3:1) has been observed, and the majority of cases are under 65 years of age [6,7]. The five-year survival rate is reported to be around 14%. Familial variants of this malignancy with even more fatality (mean survival rate: four months) have also been reported [8,9].

Primary cardiac angiosarcoma is a clinically significant condition due to its rarity and aggressive behavior. It has a poor prognosis. Rapid development and early spread of the tumor result in advanced illness at the time of diagnosis. Primary cardiac angiosarcoma is thus linked to significant death rates. The aggressive nature of primary cardiac angiosarcoma is attributed to its propensity for early metastasis to distant sites, such as the lungs, liver, and lymph nodes. Curative therapy is difficult since metastatic dissemination is frequently seen before the tumor in the heart is discovered. Furthermore, a full surgical resection is challenging due to the tumor's infiltrative nature, and even after vigorous treatments, the tumor frequently returns [10].

Primary cardiac angiosarcoma presents unique diagnostic and treatment problems due to its intricacy and rarity. It is necessary to develop new, effective methods to improve the early detection, accurate diagnosis, and optimum treatment of this aggressive malignancy. The objective of this article is to provide a comprehensive analysis of primary cardiac angiosarcoma, including details on its pathogenesis, clinical presentation, current treatments, prognosis, and follow-up. This review aims to summarize the current knowledge and understanding of primary cardiac angiosarcoma by examining the existing literature and research while also highlighting gaps and areas that require more investigation. This narrative review aims to advance the knowledge of this uncommon and aggressive malignancy and pave the path for future advancements in diagnostic and treatment approaches.

## Review

Pathogenesis and molecular biology

Histopathology and Immunohistochemistry

**Figure 1:**
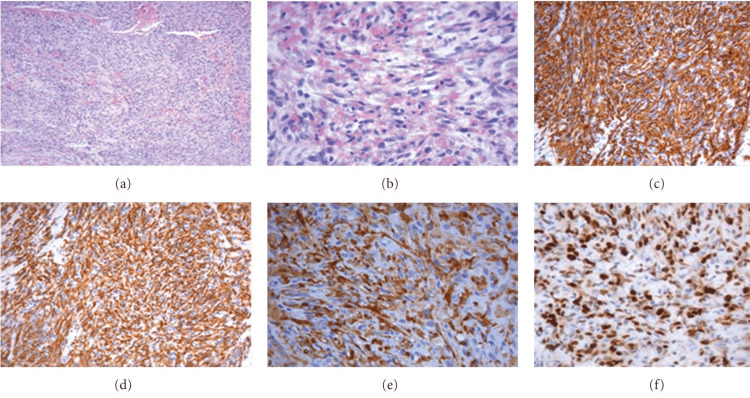
(a) Angiosarcoma moderately differentiated (coloring H&E, 10×). (b) Greater enlargement angiosarcoma moderately differentiated (coloring H&E, 40×). (c) Tumor cells were positive for CD31 (immunohistochemical investigation, 20×). (d) Tumor cells were positive for CD34 (immunohistochemical investigation, 20×). (e) Tumor cells were positive for FVIII rAg (immunohistochemical investigation, 20×). (f) Positive for Mib-1 60% (40×). Image adapted from Antonuzzo et al. [6] FVIII rAg: factor VIII-related antigen

Histopathological examination and immunohistochemistry play crucial roles in the diagnosis of primary cardiac angiosarcoma. The histological diagnosis of these tumors can be challenging, with a low success rate in obtaining a diagnosis through pericardial fluid analysis, endomyocardial biopsies, and pericardial biopsies [11]. Microscopic visualization reveals the presence of anastomotic vascular channels formed by malignant cells, solid areas of spindle cells, and other areas of primarily anaplastic cells (Figure 1). Histological features include anastomosing vascular channels, solid spindle cell areas, foci of endothelial tufting, and a lack of calcification [12].

Immunohistochemical stains are used to identify the endothelial origin of the sarcoma when the primary location is in question. The commonly used markers for identifying endothelial angiosarcomas include CD31, CD34, factor VIII-related protein, von Willebrand factor, cytokeratin, vimentin, BNH9, p53, Ki67, alpha-smooth muscle actin, and Wilms' tumor 1 [13-15].

CD31 and FLI-1 are highly sensitive markers for both benign and malignant vascular tumors, while CD34 is also useful but less sensitive. Positive staining for CD31 and CD34 demonstrates the presence of vascular lumens throughout the lesion [16]. Von Willebrand factor is expressed but is considered the least sensitive marker, especially in inadequately differentiated tumors. Cytokeratin shows weak focal staining in approximately one-third of angiosarcomas, and vimentin is positive in most endothelial angiosarcomas but is also positive in other cellular lineages [17].

BNH9, a monoclonal antibody against blood group-related H and Y antigens, shows high positivity in angiosarcomas but is negative for most other soft tissue sarcomas [18]. Ki67 staining, which determines the proliferation rate, is often positive and correlated with poor survival outcomes [19]. Alpha-smooth muscle actin staining is positive in the vessels and pericytes surrounding neoplastic endothelial cells in cardiac angiosarcomas [17]. Wilms' tumor 1 staining is helpful in distinguishing cardiac angiosarcomas from other types of cardiac sarcomas [19].

In summary, histopathological examination reveals distinct histological features of primary cardiac angiosarcoma, including anastomosing vascular channels and spindle cell areas. Immunohistochemistry, with a combination of endothelial markers such as CD31, CD34, and factor VIII-related protein, aids in confirming the endothelial origin of the tumor [16]. These techniques play a crucial role in differentiating angiosarcomas from other soft tissue neoplasms and in establishing an accurate diagnosis of primary cardiac angiosarcoma.

Molecular Alterations and Genetic Factors

One of the notable molecular alterations detected in cardiac angiosarcoma involves the genes kinase insert domain receptor (*KDR*), *KIT*, and cyclin-dependent kinase inhibitor 2A (*CDKN2A*) [20,21]. Soft tissue angiosarcomas typically have mutations in kinase insert domain receptor (*KDR*), which encodes one of the vascular endothelial growth factor receptor tyrosine kinases. *KDR* mutations have been found in the transmembrane or immunoglobulin domains of the protein in some cases of cardiac angiosarcoma and are present in about 7%-10% of soft tissue angiosarcomas [22]. Another commonly altered gene in soft tissue angiosarcomas is phospholipase C gamma 1 (*PLCG1*), which is associated with the phosphoinositide signaling system. Cardiac angiosarcoma cases have been found to have mutations in *PLCG1*, predominantly affecting the auto-inhibitory Src homology 2 (cSH2) domain within exon 18 [23]. These mutations can confer primary resistance against therapies targeting the vascular endothelial growth factor (VEGF)/KDR pathway [24,25].

Primary cardiac angiosarcomas seldom exhibit myelocytomatosis oncogene (*MYC*) amplification, a genetic change that is frequent in soft tissue angiosarcomas [26,27]. Angiosarcomas, particularly cardiac angiosarcoma, rarely undergo tumor protein p53 (*TP53*) alterations that have been seen in sarcomas with complicated karyotypes [27,28]. A subgroup of angiosarcomas, including a few cases of cardiac angiosarcoma, has also been shown to contain RAS pathway abnormalities, such as mutations in H/K/N-RAS and KRAS [26,27]. Several genetic alterations commonly observed in soft tissue angiosarcomas, such as Capicua transcriptional repressor (*CIC*) fusions and mutations, phosphatidylinositol-4,5-bisphosphate 3-kinase, catalytic subunit alpha (*PIK3CA*), Fms-related tyrosine kinase 4 (*FLT4*), and tyrosine kinase with immunoglobulin-like and EGF-like domains 1 (*TIE1*), have not yet been investigated in cardiac angiosarcoma [24,29].

Particular mutations have been found only in cardiac angiosarcoma. For example, inactivating mutations of the *KMT2D* (lysine methyltransferase 2D) gene have only been found in extra-cardiac angiosarcomas [24]. In addition, it has been shown that changes in the protection of telomeres 1 (*POT1*) gene have been found in a subgroup of Li-Fraumeni-like families and sporadic cardiac angiosarcoma patients [30]. Both familial and sporadic cardiac angiosarcoma patients have shown *POT1* mutations, and their functional ramifications are now being investigated.

Improving diagnostic and treatment methods requires a better understanding of the molecular changes and genetic variables involved in the development of cardiac angiosarcoma. Potential therapy for this aggressive malignancy may include targeted medications that target certain molecular abnormalities. Further study is required to decipher the intricate molecular landscape of cardiac angiosarcoma and find more genetic mutations and therapeutic options.

Angiogenesis and Vascular Endothelial Growth Factor (VEGF)

Angiogenesis, the formation of new blood vessels from pre-existing ones, is a complex process involving various cellular and molecular interactions. In the context of cardiac angiosarcoma, angiogenesis plays a crucial role in tumor development and progression.

There are multiple sequential phases that make up the angiogenic response in the microvasculature. Proteases first break down the basement membrane. Then, endothelial cells (ECs) proliferate at the migratory tip after sprouting in the interstitial space. The development of a new basement membrane, the recruitment of pericytes, the anastomosis with existing blood vessels, and the establishment of blood flow are all components in the production of new blood vessels. During neovascularization, the extracellular matrix (ECM), ECs, and pericytes' adhesion contacts change due to the rearrangement of the cytoskeleton, production of cell surface adhesion molecules, secretion of proteolytic enzymes, and modification of the ECM [31].

Angiogenesis in tumors, including cardiac angiosarcoma, is driven by a switch to an angiogenic phenotype. Tumor cells can overexpress angiogenic growth factors such as vascular endothelial growth factor (VEGF) and basic fibroblast growth factor (bFGF), mobilize angiogenic proteins from the ECM, and recruit host cells such as macrophages that produce angiogenic factors or engage in a combination of these processes. Tumor-secreted angiogenic growth factors interact with their receptors on ECs, initiating a cascade of signaling events [32]. VEGF, a highly expressed angiogenic growth factor in various tumors, binds to its receptors (Flt-1/VEGFR-1 and Flk-1/KDR/VEGFR-2) on ECs, leading to the dimerization of receptors and activation of downstream signaling proteins [33]. These signaling pathways, including PI3-kinase, Src, Grb2/m-SOS-1, and STATs, regulate the cell cycle machinery and promote EC proliferation [34].

The balance between positive and negative regulators of microvessel growth determines the angiogenic phenotype. An excess of angiogenic factors in the tumor microenvironment promotes the persistence and maturation of neovessels. Conversely, an abundance of angiostatic factors can induce neovessel regression by inducing apoptosis or cell cycle arrest in ECs. Therefore, the local equilibrium between positive and negative regulators plays a critical role in the angiogenic switch [34].

VEGF is a key player in tumor angiogenesis and acts as an endothelial-specific mitogen. It enhances EC permeability, promotes EC proliferation through autophosphorylation of receptors, and induces the formation of second messengers. VEGF also stimulates proteolysis and remodeling of the ECM, allowing EC migration and vessel formation. It activates various signaling pathways, including the mitogen-activated protein (MAP) kinase cascade and the phosphatidylinositol 3‑kinase (PI3K)/protein kinase B (Akt) pathway, promoting EC survival, migration, and proliferation. VEGF also regulates the expression of integrins involved in cell migration and matrix reorganization [35,36].

In cardiac angiosarcoma, VEGF and its receptors play crucial roles in tumor angiogenesis. VEGF expression is upregulated, leading to increased vascular permeability, the proliferation of EC, and the formation of new vessels. The autocrine and paracrine effects of VEGF contribute to the survival and growth of ECs in newly formed immature vessels [37]. Other angiogenic factors, such as angiopoietin-1 and αvβ3-integrins, also contribute to EC survival and vessel formation in cardiac angiosarcoma [34,35].

Understanding the angiogenic mechanisms in cardiac angiosarcoma provides insights into potential therapeutic targets. Targeting the VEGF pathway and other key angiogenic factors may offer new treatment strategies for this aggressive malignancy. Further research is needed to uncover the specific molecular mechanisms underlying angiogenesis in cardiac angiosarcoma and identify additional therapeutic targets.

Clinical presentation

Primary cardiac angiosarcoma is a rare and aggressive tumor that presents with a variety of symptoms and signs. Due to its location within the heart, the clinical presentation of cardiac angiosarcoma can be diverse and nonspecific, often leading to delayed diagnosis.

The most common symptom reported by patients with cardiac angiosarcoma is dyspnea (59%-88%), which is present in the majority of cases [1]. This symptom is often attributed to the tumor's obstructive effects on blood flow within the heart, resulting in congestive heart failure. Patients may also experience fatigue, chest pain, palpitations, and syncope. These symptoms are indicative of the tumor's impact on cardiac function and may be related to tumor size, location, and invasiveness [38].

Other clinical manifestations of cardiac angiosarcoma can include pericardial effusion, cardiac tamponade, and valvular dysfunction. The tumor's infiltrative nature can lead to the involvement of neighboring structures, such as the pericardium and cardiac valves, causing compression, effusion, and regurgitation [39]. Consequently, patients may present with symptoms related to fluid accumulation or compromised cardiac function.

In some cases, patients with cardiac angiosarcoma may develop systemic symptoms due to metastasis. These symptoms can include weight loss, anemia, and generalized malaise. Metastases commonly occur in the lungs, liver, lymph nodes, bones, and adrenals, and their presence can significantly worsen the prognosis of the disease [40-42].

Diagnostics and imaging modalities

The diagnosis of primary cardiac angiosarcoma can be challenging due to its rarity and nonspecific clinical presentation. Several diagnostic methods and imaging techniques are employed to aid in the early detection and accurate diagnosis of this aggressive tumor.

Echocardiography is often the initial imaging modality used to evaluate patients with suspected cardiac tumors because it is a widely available, inexpensive, and noninvasive procedure. Transesophageal echocardiography (TEE) is frequently used as the first line of diagnostics and has a sensitivity of 97% for identifying cardiac masses [43]. It allows for the assessment of tumor location, size, and mobility and the presence of associated complications, such as pericardial effusion or valvular abnormalities [44]. Echocardiography can provide valuable information to guide further diagnostic workup and treatment planning. The limitations of echocardiography include its difficulty in accurately describing various tissue types and its reliance on the skill and technique of the operator [43]. However, if the patient is above 50 years of age, coronary arteriography may be recommended to evaluate the coronary arteries [45].

Metastases are often widespread at the time of diagnosis, with the lungs being the most common site of metastatic disease [10]. CT scans can provide a better understanding of the cardiac tumor anatomy and help detect systemic metastasis. It also aids in visualizing calcifications associated with the tumor and helps in transthoracic biopsy [1]. Cardiac MRI is superior to CT in characterizing soft tissue and distinguishing between different abnormalities specific to the myocardium. It can help differentiate between thrombi and tumors in the cardiac cavity [46]. MRI may show distinct patterns for angiosarcoma, including areas of increased signal intensity dispersed among areas of low to intermediate signal intensity, giving a cauliflower-like appearance, or linear contrast material enhancement along vascular pools demonstrating a sunray appearance [47]. However, motion artifacts may be encountered with cardiac MRI [46].

A tissue biopsy is essential for confirming cardiac angiosarcoma. Pericardiocentesis and pericardial fluid cytology are unreliable methods, with malignant cells rarely found even when the tumor has invaded the pericardium [11]. Endomyocardial biopsies are also not effective in diagnosing cardiac angiosarcoma, with a low diagnostic yield [48]. For an accurate diagnosis, surgical exploration and intraoperative frozen sections are recommended. Open cardiac biopsy or surgical resection for tissue diagnosis is often performed, particularly if the tumor is located in the right atrium [49].

The differential diagnosis for cardiac angiosarcoma is broad and includes thrombus, vegetation, foreign body, intracardiac metastases, infectious and nonbacterial thrombotic or marantic endocarditis, coronary artery disease, constrictive cardiomyopathy, and other malignancies such as bronchogenic carcinoma or mesothelioma [50].

In summary, the diagnosis of cardiac angiosarcoma involves a combination of imaging techniques such as echocardiography, CT, and MRI. Tissue diagnosis through surgical exploration and histopathological evaluation is crucial for confirming the presence of angiosarcoma. Differential diagnosis is essential to rule out other cardiac conditions and malignancies. Prompt and accurate diagnosis is challenging due to the rarity of the disease and its often advanced stage at presentation.

Treatment approaches

The primary treatment approach for primary cardiac angiosarcoma is surgical resection with the goal of achieving complete tumor removal. Complete resection offers the best chance of long-term survival and potentially curative outcomes [51]. The extent of surgical resection depends on the location and size of the tumor, as well as the involvement of adjacent structures. The surgical techniques used may involve excision of the tumor along with partial or complete removal of the affected chamber(s) of the heart. In some cases, reconstruction of the cardiac structures may be necessary to restore normal function [10,52].

Surgical management of primary cardiac angiosarcoma poses several challenges and limitations. Due to the infiltrative nature of the tumor, achieving complete resection can be difficult, especially when the tumor has spread to adjacent structures or metastasized. Additionally, the heart's delicate anatomy and vital functions require careful surgical planning to minimize the risk of complications such as bleeding, cardiac dysfunction, or postoperative arrhythmias [1,53]. In some cases, the tumor may be inoperable due to extensive involvement or distant metastasis. Surgical treatment should be carefully considered in conjunction with a multidisciplinary team to assess the feasibility and potential benefits of the procedure [54].

Adjuvant chemotherapy is commonly administered following surgical resection of primary cardiac angiosarcoma [55]. The choice of chemotherapy regimen depends on factors such as tumor stage, patient's overall health, and individualized treatment plans. The most commonly used chemotherapy agents include anthracyclines (e.g., doxorubicin), taxanes (e.g., paclitaxel), and ifosfamide. Combination chemotherapy regimens, such as the MAID (mesna, doxorubicin, ifosfamide, and dacarbazine) or AIM (doxorubicin, ifosfamide, and mesna) protocols, have been employed to improve treatment efficacy [56-58]. However, the optimal chemotherapy regimen for primary cardiac angiosarcoma has not been definitively established, and treatment outcomes remain modest.

Radiation therapy may be utilized as part of the treatment approach for primary cardiac angiosarcoma, either as adjuvant therapy after surgery or as definitive therapy in cases where complete surgical resection is not feasible [59]. The aim of radiation therapy is to achieve local control and reduce the risk of local recurrence. External beam radiation therapy is commonly employed, delivering high-energy radiation to the tumor site while sparing adjacent normal tissues. However, the heart's critical structures and proximity to radiation fields pose challenges in delivering effective doses without causing significant cardiac toxicity [60]. Therefore, radiation therapy should be carefully planned and individualized based on the patient's specific circumstances.

Given the complexity and rarity of primary cardiac angiosarcoma, a multidisciplinary approach involving collaboration among various specialties is crucial in the management of the disease. A multidisciplinary team typically includes cardiothoracic surgeons, medical oncologists, radiation oncologists, radiologists, pathologists, and specialized nurses. The team collaborates to develop an individualized treatment plan based on the patient's specific condition, tumor characteristics, and treatment goals.

Close communication and coordination among team members are essential to ensure optimal treatment outcomes. The team discusses treatment options, assesses the feasibility of surgical resection, determines the need for adjuvant therapies, and monitors the patient's response to treatment. Regular tumor board meetings and case discussions facilitate the exchange of expertise and allow for shared decision-making. Throughout the treatment process, specialized nurses and supportive care teams play a crucial role in managing the patient's physical and emotional well-being. They provide education, symptom management, and supportive care to optimize the patient's quality of life.

## Conclusions

Primary cardiac angiosarcoma is a rare and aggressive malignancy originating from the endothelial lining of cardiac blood vessels. It presents significant challenges in diagnosis and treatment due to its infiltrative nature and early metastasis. Echocardiography, along with imaging techniques such as CT and MRI, plays a crucial role in the diagnosis and assessment of the tumor. Histopathological examination and immunohistochemistry are essential for confirming the diagnosis, with specific endothelial markers aiding in differentiation from other soft tissue neoplasms. Early detection of primary cardiac angiosarcoma remains challenging due to its rarity, and the prognosis is generally poor due to advanced disease at the time of diagnosis. A multidisciplinary approach and collaboration among different specialties are emphasized to optimize diagnosis, treatment, and follow-up care. Further research is needed to enhance diagnostic methods and therapeutic approaches for this aggressive malignancy. Ultimately, advancing knowledge and understanding of primary cardiac angiosarcoma will pave the way for improved outcomes and better management of this challenging condition.
